# Crosstalk-Free Excitation Scheme for Quantitative OH Laser-Induced Fluorescence in Environments Containing Excited CO

**DOI:** 10.1177/00037028221088591

**Published:** 2022-04-25

**Authors:** Maik Budde, Richard Engeln

**Affiliations:** 1 Department of Applied Physics, 3169 Eindhoven University of Technology, Eindhoven, The Netherlands; 2 3169 Instituto de Plasmas e Fusão Nuclear, Instituto Superior Técnico, 3169 Universidade de Lisboa, Portugal

**Keywords:** Laser-induced fluorescence spectroscopy, LIF, OH, CO third-positive system, CO2–H^2^O plasma

## Abstract

Spectral overlap in the single-photon laser-induced fluorescence between the 3064 Å system of OH and the third positive system of CO is detected in a highly-excited environment, namely, a CO_2_-H_2_O plasma. The overlap is distorting excitation and fluorescence spectra as well as fluorescence time decays of commonly used excitation transitions of OH. As a consequence, systematic errors are introduced into the determination of temperatures, gas compositions, and absolute number densities. The P_1_(2) transition is proposed to circumvent the distortion while still allowing for quantitative measurements due to the availability of non-radiative rate coefficients.

## Graphical abstract

**Graphical Abstract:**
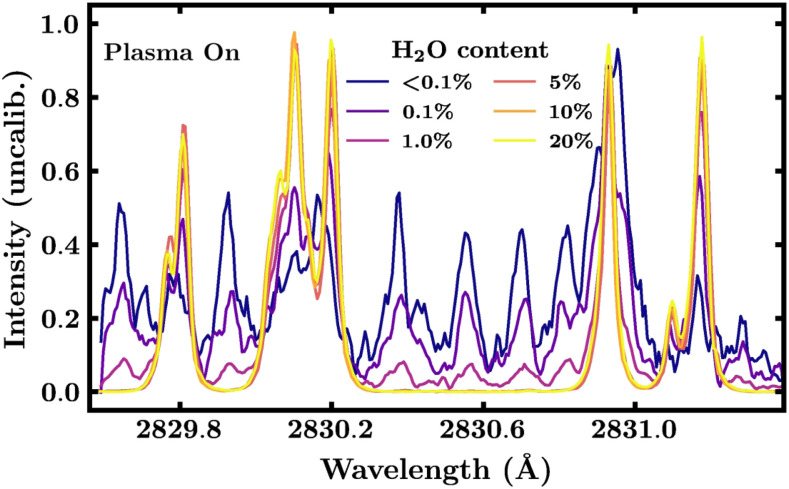

## Introduction

The hydroxyl radical OH is relevant in various fields of research, e.g. from atmospheric processes^1^ and plasma medicine^2^ over to combustion^3^ and plasma conversion,^4^ due to its high reactivity. Among the diagnostic techniques used to detect OH, laser-induced fluorescence (LIF) spectroscopy has been proven to be a powerful method to measure OH space- and time-resolved even in challenging environments.^5–7^

Besides absolute number densities, in case of a proper calibration, further quantitative measurements like the conversion of an input gas or the rotational temperature of OH are possible.^4,8^ The quantification of LIF experiments is based on the simulation of experimental outcomes like the time-dependent decay of the emitted fluorescence after laser excitation, that is, the fluorescence pulse, or the spectrally resolved fluorescence spectrum.^9–11^ Models used for that purpose delicately rely on the used state-to-state-dependent rate coefficients for collisional processes.

Particularly, OH LIF is a special case since commonly used exciting lasers with nanosecond pulse duration have narrow band widths allowing to populate a single rotational level in the electronically excited state.^12^ This nascent rotational distribution can become a Boltzmann distribution if redistribution across the other rotational levels by a sufficient number of rotational energy transfer collisions occurs before the excited state is depopulated by (non-)radiative processes. Depending on whether the rotational distribution is thermalized, that is, Boltzmann, or not yet, thermal^13^ or non-thermal^12^ rate coefficients must be used, due to the state-to-state dependence of these coefficients. In conclusion, an important aspect in the selection of a LIF excitation scheme, when striving for quantitative measurement, is the availability of (non-)thermal rate coefficients.

Another crucial consideration in the selection of a certain transition for excitation or as observable in the experiment is potential overlap with the absorption or emission of other (excited) species, respectively. Usually, such overlap is complicating the analysis of data and must thus be circumvented if possible, for example, by selecting another transition.

One molecule that is often encountered together with OH is carbon monoxide, for example, in combustion^3^ or CO_2_ plasma conversion research.^5,14^ Their coexistence is not only important from a chemical point of view, for example, due to their exothermic reaction CO+OH → CO_2_+H^15^ but also from a spectroscopic point of view, that is, due to the spectral overlap in particular excitation schemes of both species. The spectral overlap in the excitation of the 3064 Å system of OH (single-photon) and the fourth positive system of CO (two-photon) with laser radiation around 283 nm is known. Nevertheless, the different resulting fluorescence wavelengths permit crosstalk-free measurements of both molecules with the same laser.^3,16^ Similar issues with other CO systems are not discussed in literature. In particular, studies on laser excitation of the CO third positive system (TPS) CO{a^3^Π}↔CO{b^3^Σ^+^} are scarce. The available studies tend to report single-photon LIF on the TPS even though the lower triplet state CO{a^3^Π} is populated through absorption of at least one more laser photon beforehand, for example, in 2+1 multiphoton ionisation experiments.^17,18^

In the present study, we demonstrate spectral crosstalk between the 3064 Å system of OH and the TPS of CO not only in the excitation wavelength, that is, around the P_1_(3) line of OH at 2830.93 Å but also in the wavelength range of detection, that is, from 305 nm to 320 nm containing the (0,0) and the (1,1) band,^9^ and the systematic error introduced with improper consideration of the overlap. Furthermore, real single-photon LIF on the CO TPS is shown because the CO{a^3^Π} population apparently is sufficient without the need for an additional pumping laser. Eventually, a method to still measure OH quantitatively without spectral crosstalk is proposed. For clarity throughout this letter we use curly braces { } for states and transitions of CO while brackets ( ) are used for OH. The given wavelengths are vacuum wavelengths.

### Demonstration of the Spectral Overlap Between OH and CO

**Figure 1.**
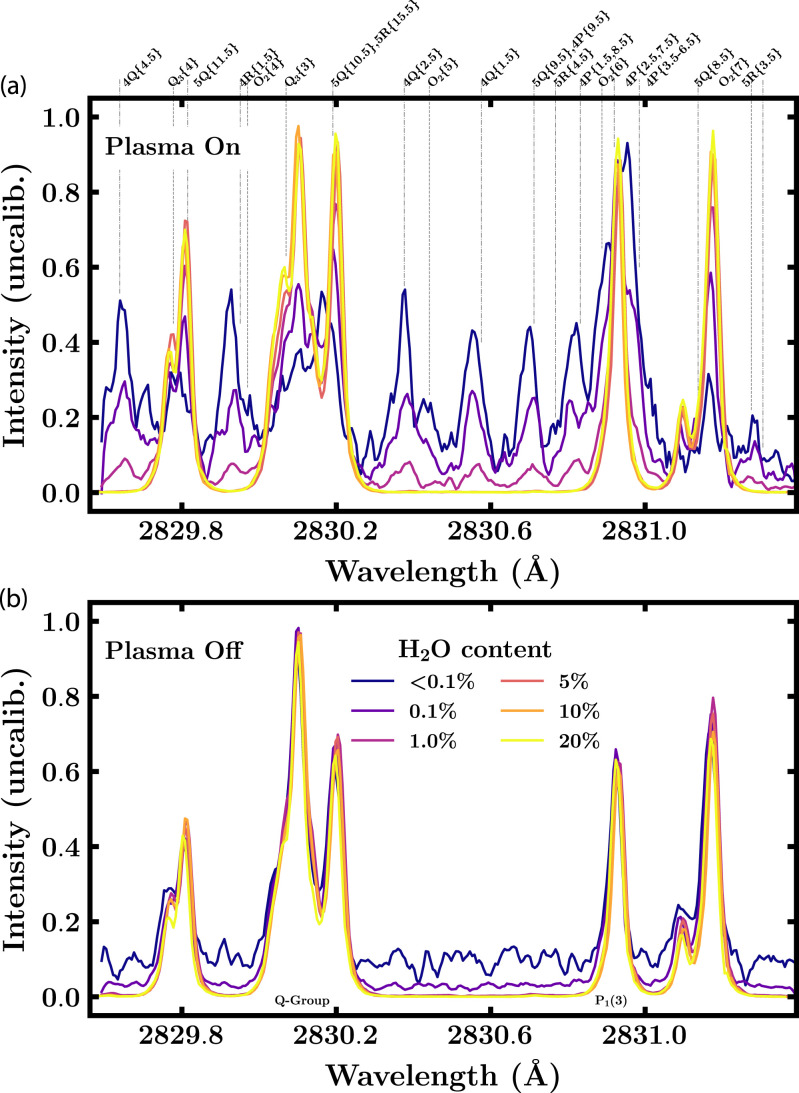
Excitation spectra for conditions with different amounts of water admixed to the CO_2_ glow discharge. The laser is fired while the plasma is on a) or when it is off b). The labels at the bottom of b) indicate conventionally used OH transitions. Transitions of the CO TPS are identified in (a).^27,28^

In Fig. 1, the excitation spectra are shown. These spectra are recorded in a glow discharge in CO_2_ to which different amounts of water are added. The setup used to record these spectra has been described in detail elsewhere;^19^ hence only the most important components, namely, the exciting laser and the plasma source, are recapped here. The exciting laser is a dye laser (Sirah Cobra Stretch) filled with rhodamine 590 dye dissolved in ethanol which is pumped by the second harmonic of a neodymium-doped yttrium aluminum garnet (Nd:YAG) laser (Spectra Physics Quanta Ray Pro 290–30). The resulting laser radiation is frequency-doubled to obtain tunable radiation around 283 nm. The laser light passes in axial direction through the glow discharge reactor that consists of a 23 cm long Pyrex tube with 2 cm inner diameter. A plasma is ignited by applying a pulsed voltage (5 ms on, 11.67 ms off) with a high-voltage amplifier (Trek 10/40A-HS) yielding a discharge current of 50 mA. The pressure in the reactor is kept constant at 6.67 mbar by providing CO_2_ gas (Linde 4.5 Instrument, 99.995%) through mass flow controllers (Bronkhorst F-201CV) either in dry condition or enriched with water by guiding the gas stream through a controlled evaporator mixer (Bronkhorst W-101A). By mixing dry and humid gas streams different water admixtures are realized. Fluorescence is collected under a 90° angle with respect to the laser, dispersed in a spectrometer (Andor Shamrock SR-303i) and detected temporally resolved with a photomultiplier tube (PMT) (Hamamatsu H11526-20-NF).

The distortion of the OH laser-induced fluorescence signal is shown with the aid of the excitation spectra in Fig. 1. The spectra are obtained by scanning the excitation laser wavelength while keeping the detection window, selected with the spectrometer, constant around 305 nm–320 nm. For each laser wavelength the time-dependent fluorescence pulse is measured with the PMT. Integration of the pulse over time gives the corresponding point in the excitation spectrum.

In Fig. 1 the amount of admixed water is changed from 20% to 0.1%. An admixture of < 0.1% corresponds to residual water. The results of the measurements presented in this letter are not aiming for quantitative reproducibility but for illustrating the spectral distortion which is also why signal strength is favored over maintaining LIF linearity with laser energy per pulse. With small amounts of residual water it is fortunately still possible to identify the underlying excitation spectra of OH. The scaling and smoothing (second-order Savitzky–Golay with seven-point window) of the spectra facilitates the comparison of the overall spectral shape.

For the measurements in Fig. 1a, the delay between laser shot and plasma pulse is set such that the plasma is on when the laser is fired. Hence, electron impact can still lead to excitation and formation of species. On the other hand, all measurements in Fig. 1b are obtained while the plasma is off. Thus, only species with a lifetime in the millisecond range, like OH apparently, can be observed.

For large water admixtures, that is, the yellow line in both figures, the plasma is clearly dominated by OH. The spectra inside and outside the plasma look nearly identical, exhibiting the clear features of an OH excitation spectrum in the temperature range below 1500 K.^20^ With decreasing water admixture many new lines appear, some of them overlapping with the OH lines. It is important to point out that the peaks in the excitation spectrum are indeed caused by interaction with the laser and are not a consequence of plasma emission. The latter would result in a baseline shift in the measured PMT signal that is corrected for in the data processing.

When comparing the newly occurring lines at lower water content with the line positions of the most prominent species in a CO_2_–H_2_O glow discharge, it turns out that these lines coincide with transitions in the third positive system of CO, see the line assignments in Fig. 1a.^21,22,27,28^ In particular, a number of CO lines is overlapping strongly with the P_1_(3) line of OH at 2830.93 Å.^9,21^ The labeling of the CO lines is not consistent between Asundi and Richardson, and Dieke and Mauchly since the former identified them as quintets while the latter argues that they are triplets.^27,28^ For the argumentation here it is sufficient though to attribute the lines to the CO TPS. Additionally, the detection range from 305 nm to 320 nm that is used to detect the (0,0) and the (1,1) band of OH overlaps with the {0,2} band head of the CO TPS at 3131.47 Å.^9,21^ The hypothesis that LIF on the CO TPS is observed, is further corroborated in the Supplemental Material.

Apparently, the used glow discharge is a quite unique environment with significant production of CO{a^3^Π} compared to other types of plasma. This would explain why the overlap with OH has, to our knowledge, not been reported in literature before, for example, in a nanosecond repetitively pulsed plasma for CO_2_ dissociation in the presence of water.^5^ This theory is supported by recent studies in a comparable glow discharge with experiments indicating the impact of CO{a^3^Π} on the behavior of the discharge^23^ and modeling results proving it.^24,25^ Nevertheless, the prominent observability of the distortion in the used glow discharge does not detract from the importance in other excited environments, where the influence of CO is less obvious.

Furthermore, Fig. 1b clearly proves that de facto single-photon LIF in the CO TPS is observed. No distortion of the excitation spectrum is noticed when the plasma is extinguished. Therefore, two-photon excitation followed by intersystem crossing as observed by Mosburger and Sick can be excluded.^16^ That process starts from the ground state and should hence be more pronounced in the plasma-off time. It must be stressed that the distortion-free fluorescence in Fig. 1b is due to the absence of CO in the triplet state, and not because there is no CO. During the residence time in the reactor molecules experience over a hundred plasma pulses thus ensuring the presence of CO at any time.^26^

### Crosstalk-Free Excitation Scheme for Quantitative OH LIF

As mentioned in the introduction, the second aspect to take into consideration when selecting an excitation transition is the availability of non-radiative rate coefficients. There are plenty of studies on thermal rate coefficients of OH^13^ (and references therein) but publications on non-thermal rate coefficients,^12^ for situations when the nascent rotational population distribution right after excitation cannot thermalize before relaxation, are scarce. For that reason, the study by Ceppelli et al. is chosen as guideline.^12^ They focus on the P_1_-branch of the (0,1) band of OH and in particular on the P_1_(3) line. Actually, that line is selected because it is well isolated. The overlap with CO reported here has supposedly not been considered before since it is less observable in environments other than our glow discharge. However, another line than P_1_(3) must be found for quantitative measurements since it clearly overlaps substantially with CO as shown above. Hence, the other P_1_ lines are tested by measuring excitation spectra and searching for a line that is not distorted when the plasma is on, i.e. in the presence of triplet CO, compared to the same measurement when the plasma is off. To get clear OH lines 0.1% of H_2_O is admixed to the CO_2_, while 0.0% is used to get a reference without OH.

**Figure 2.**
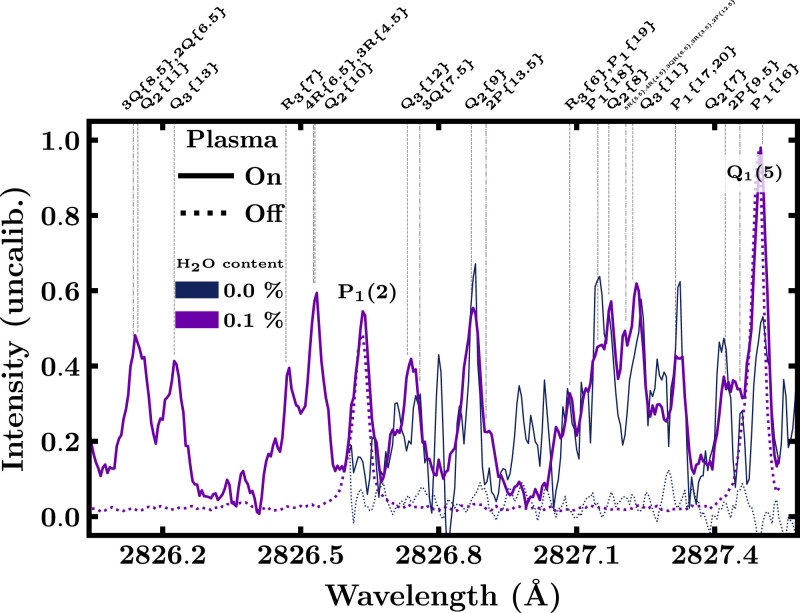
Excitation spectra around the P_1_(2) line measured in a CO_2_ glow discharge with 0.0% and 0.1% water admixture when the plasma is on (solid line) and when it is off (dotted line). Transitions of the CO TPS are identified.^27,28^

Henceforth, we propose the use of the P_1_(2) line at 2826.63 Å for quantitative LIF measurements on OH in environments that also contain CO because that line is essentially distortion-free as can be seen in Fig. 2. The absence of CO lines taken from literature^27,28^ is confirmed by the water-free measurements. Consequently, also the fluorescence spectrum is unaffected. Hence, absolute OH number densities and the gas composition can be determined by calibrated measurements of the fluorescence pulse and by collision energy transfer (CET)-LIF,^4^ respectively, without any influence of CO. For temperature determination combination with the Q_1_(5) on the right was contemplated but as can be seen in Fig. 3 that line also shows overlap with CO. Thus, we recommend the conventional scheme, that is, the Q_12_(1), Q_2_(1), Q_1_(6), Q_12_(3), and Q_2_(3) lines in the range from 2829.9 Å to 2830.3 Å that are called the *Q-group* here, for temperature determination only. An overview of the LIF schemes is given in Table 1.

**Table 1.** Comparison of the conventional OH LIF scheme with the newly proposed one. The fluorescence pulse is used to obtain absolute OH number densities, the fluorescence spectrum can be used for CET-LIF to get the gas composition,^4^ while the excitation spectrum allows for determination of the rotational temperature.^19^ The Q-Group is an abbreviation for the lines Q_12_(1), Q_2_(1), Q_1_(6), Q_12_(3), and Q_2_(3) of OH.

Scheme	Conventional	Proposed
Flu. pulse	P_1_(3)+CO	P_1_(2)
Flu. spectrum	(0,0)+(1,1) band + CO	(0,0)+(1,1) band
Exc. spectrum	Q-Group + CO	

There is a distinct trade-off between the newly proposed and the conventional excitation scheme. Non-thermal rate coefficients are only known at room temperature for P_1_(2) while for the P_1_(3) the rate coefficients can be calculated in a wide temperature range depending on the colliding species.^12^ This is only a technical obstacle though and it is the reason why for now no OH number densities can be shown (the plasma is certainly above room temperature^19^). The shown importance of the P_1_(2) might motivate others to provide non-thermal rate coefficients for that line.

## Conclusion

Calibrated laser-induced fluorescence spectroscopy on OH radicals provides valuable insights into a variety of processes, for example, through temporally and spatially resolved measurements of rotational temperatures, CO_2_ conversion fractions and absolute OH number densities in CO_2_ conversion plasmas in the presence of water.^19^ The quantification of LIF relies on (i) the availability of proper rate coefficients describing all relevant (non-)radiative processes and (ii) the quality of the spectral data.

The spectrum of a molecule is often referred to as its fingerprint allowing for the unique identification of the respective molecule. In this letter, we show that the fingerprint of OH is partially overlapping with the one of CO. In particular, the conventional excitation scheme in the 3064Å system of OH with the excitation of the P_1_(3) line for density measurements and of the excitation spectrum of the Q_12_(1), Q_2_(1), Q_1_(6), Q_12_(3), and Q_2_(3) lines for temperature determination is interfering with the third positive system of CO. Therefore, we propose the use of the P_1_(2) line for OH density and gas composition measurements, since, as we demonstrate, there is no overlap with CO for this transition. Due to the lack of other overlap-free OH transitions in the close vicinity of the P_1_(2), no new LIF thermometry scheme but the original group of lines is recommended at this point.

As a last point, it must be emphasised that the single-photon LIF on the CO TPS, which to the best of our knowledge is reported here for the first time, is not only a complication of OH LIF experiments but also an opportunity for further studies. The LIF ground state, that is, the excited triplet state CO{a^3^Π}, is of crucial importance in the presented CO_2_ glow discharge.^23–25^ Therefore, CO TPS single-photon LIF is considered a possibility to obtain a better understanding of highly-excited systems generated from CO_2_ and H_2_O mixtures as well as to validate computer models describing these systems.

## Supplemental Material

Supplemental Material - Crosstalk-Free Excitation Scheme for Quantitative OH Laser-Induced Fluorescence in Environments Containing Excited CO Click here for additional data file. Supplemental Material for Crosstalk-Free Excitation Scheme for Quantitative OH Laser-Induced Fluorescence in Environments Containing Excited CO by Maik Budde and Richard Engeln in Applied Spectroscopy.
